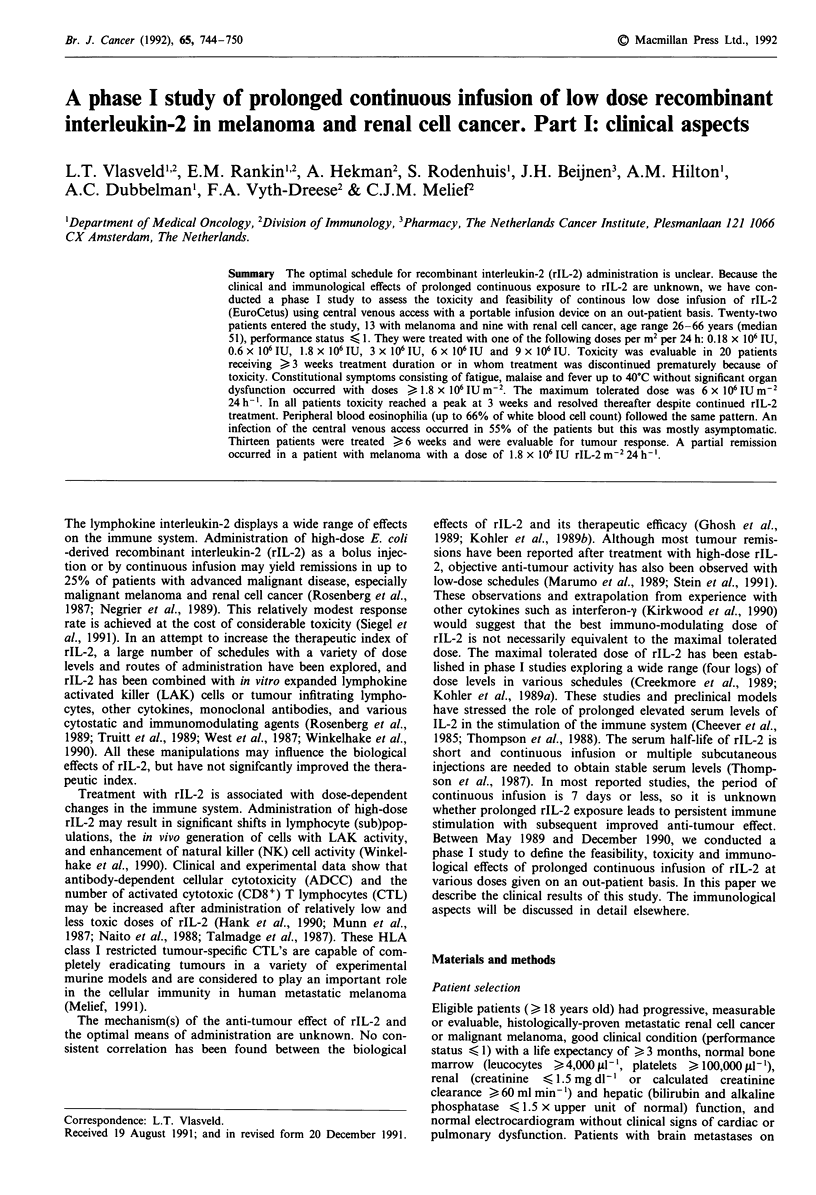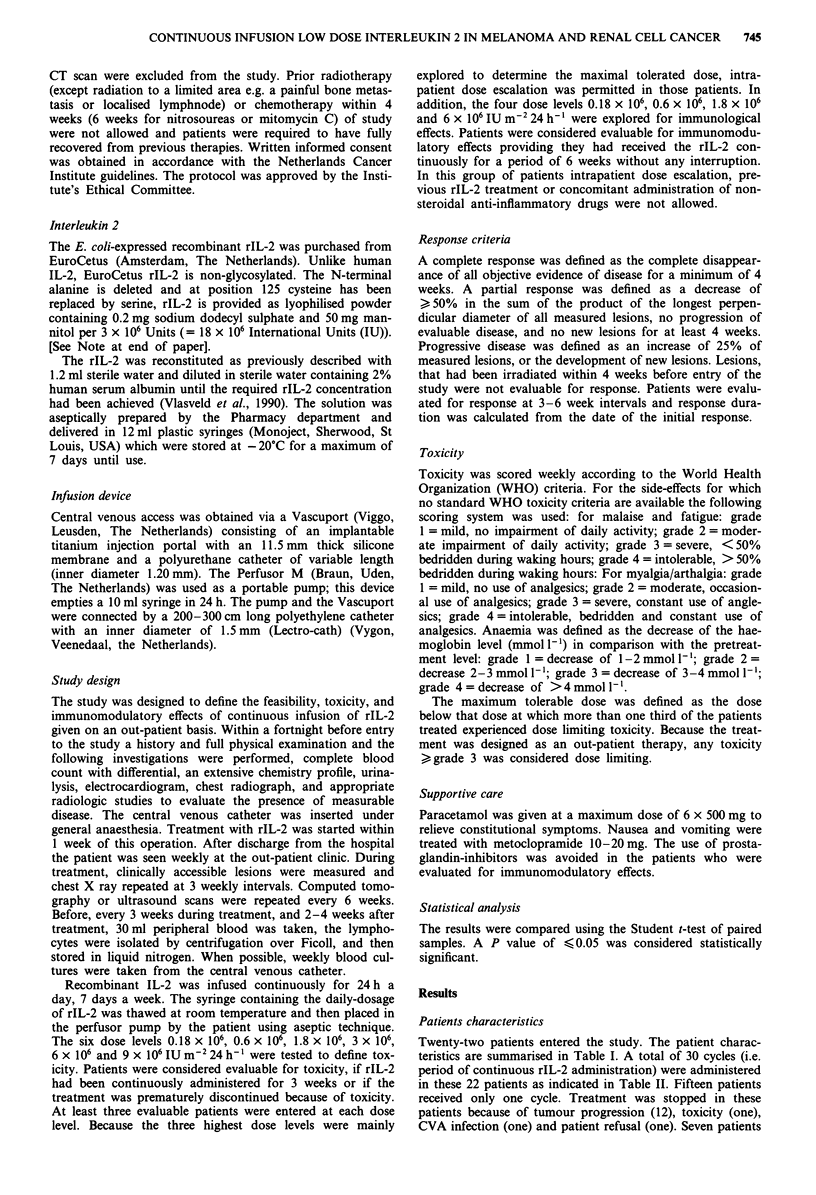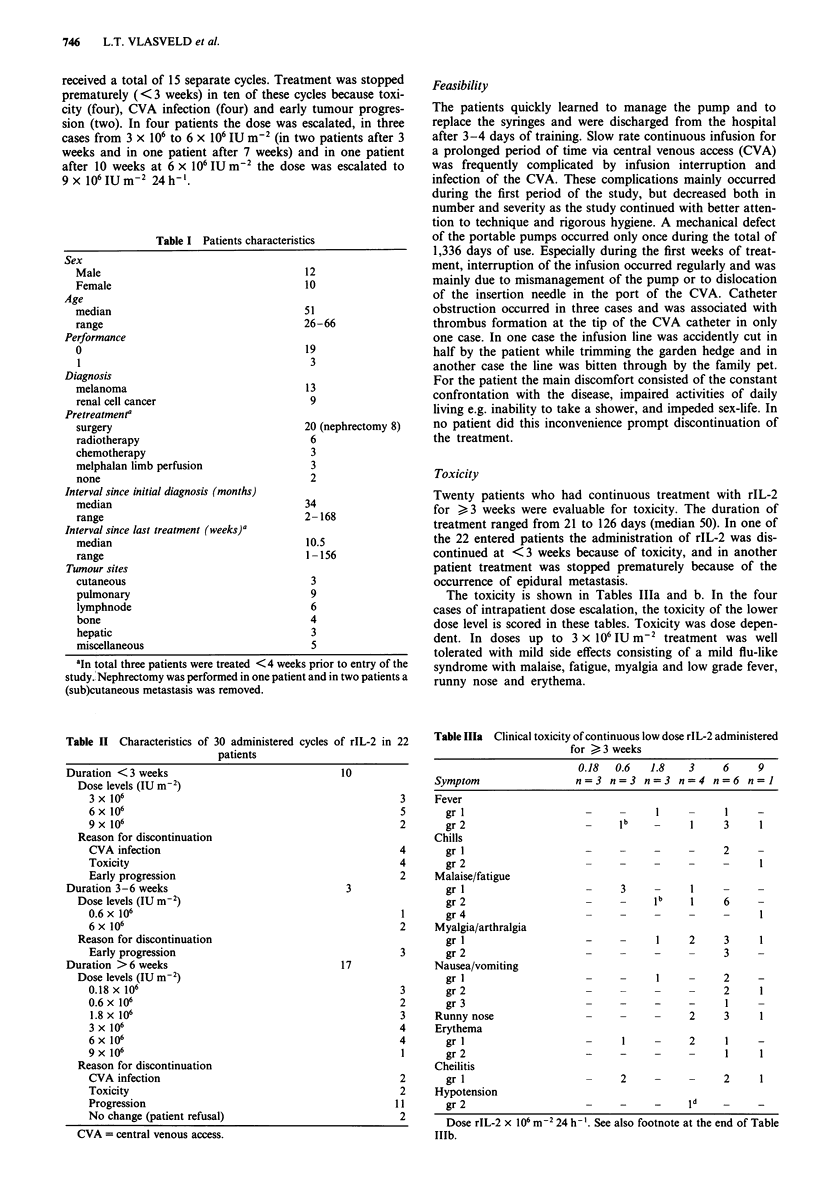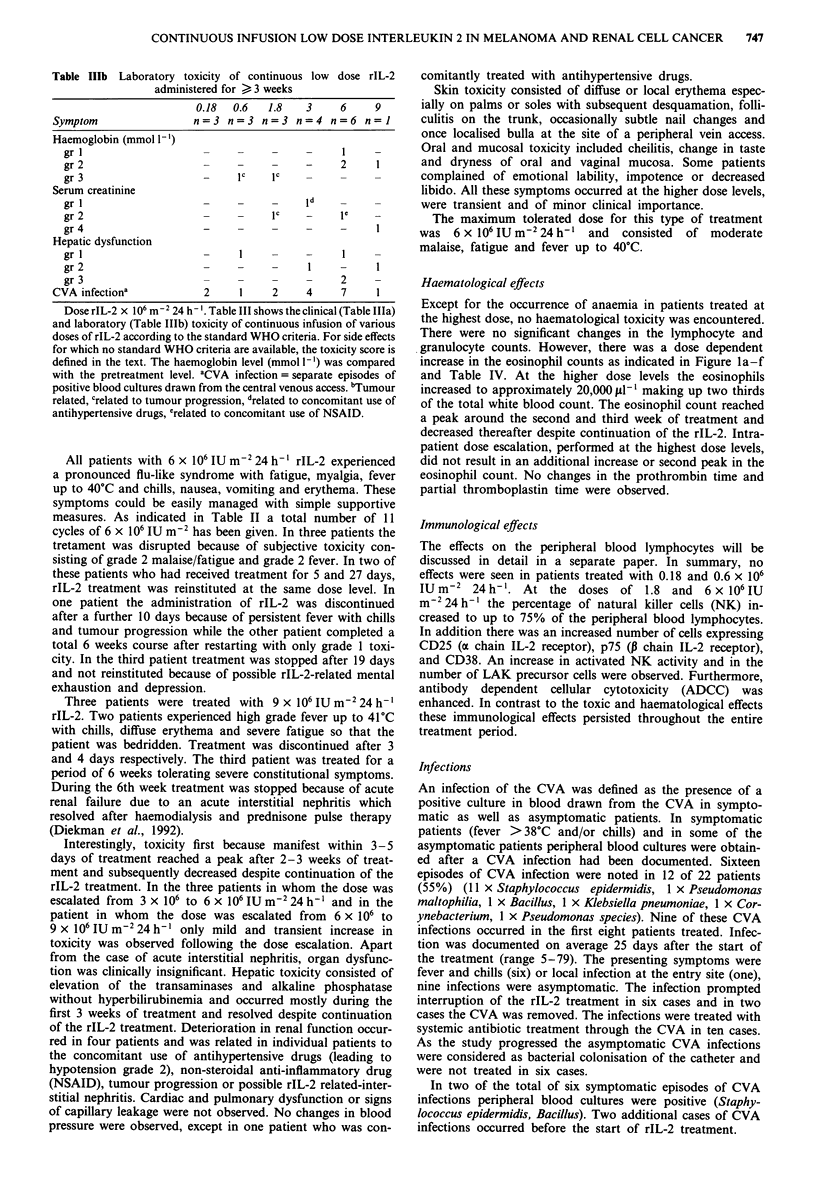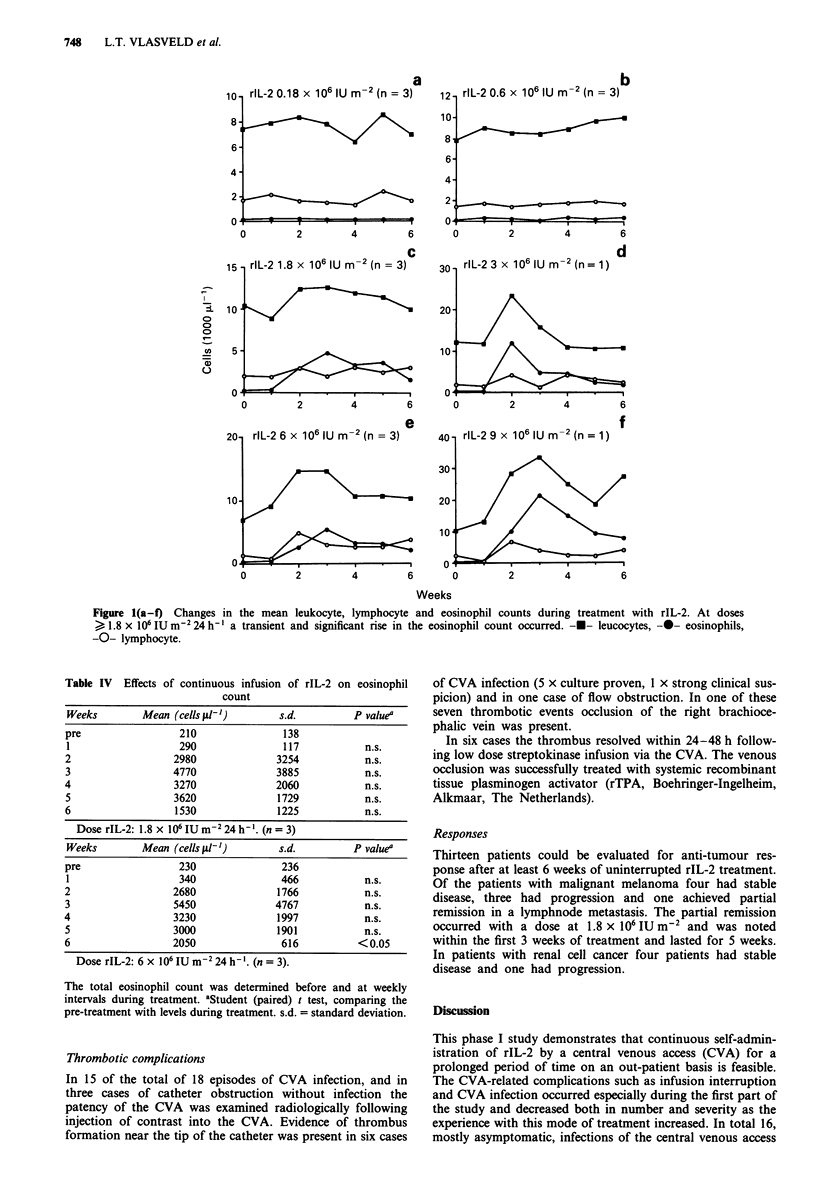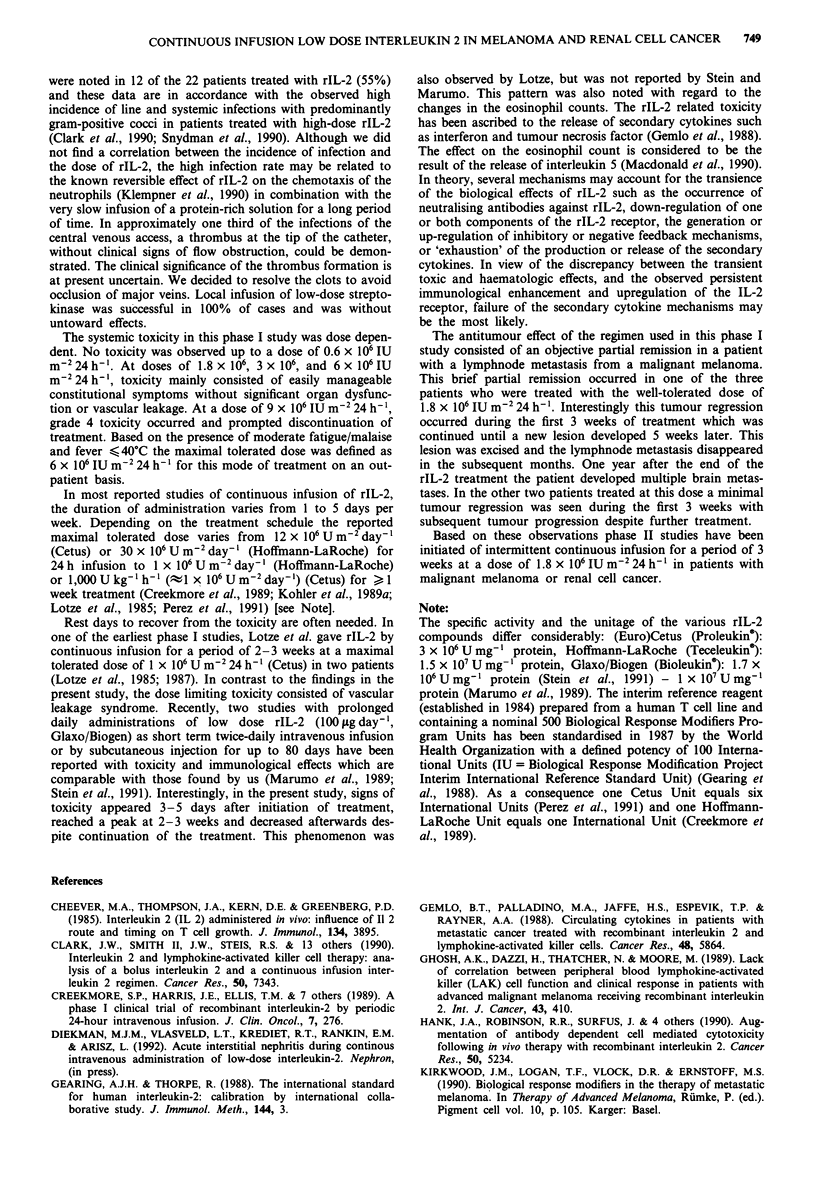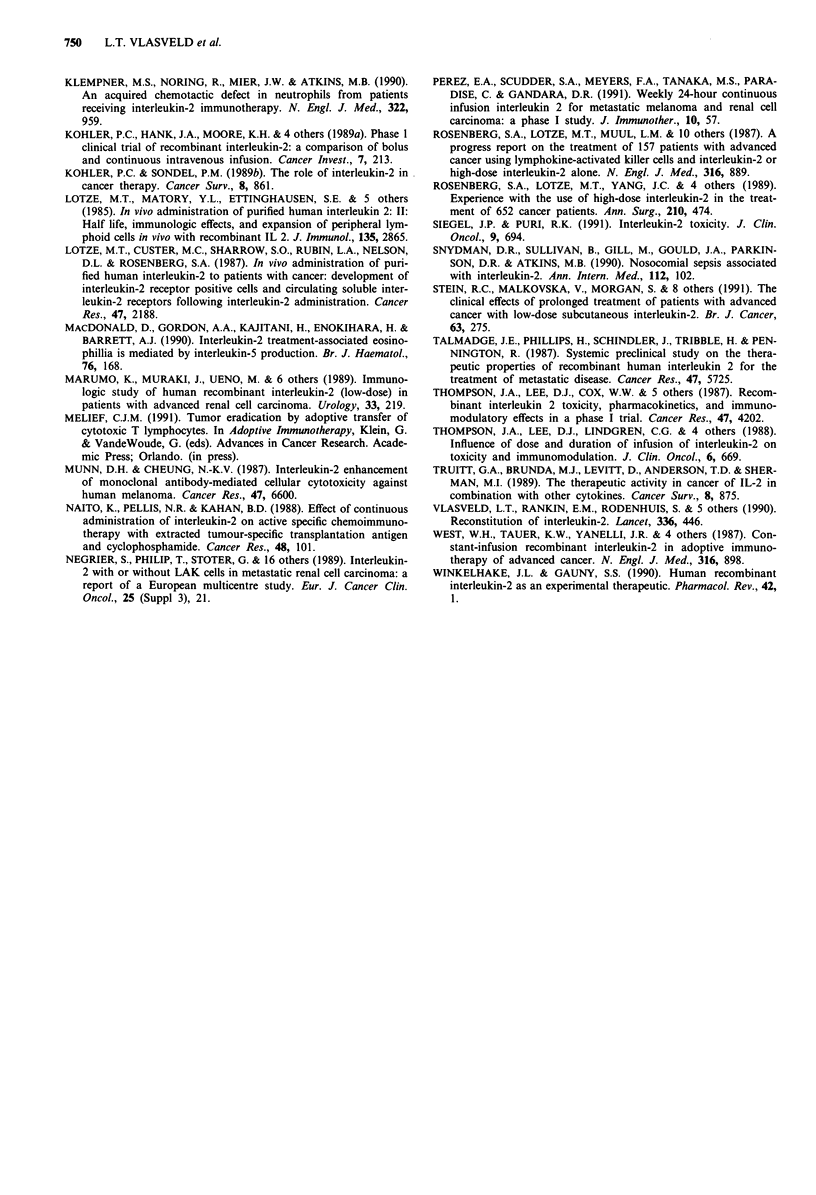# A phase I study of prolonged continuous infusion of low dose recombinant interleukin-2 in melanoma and renal cell cancer. Part I: Clinical aspects.

**DOI:** 10.1038/bjc.1992.157

**Published:** 1992-05

**Authors:** L. T. Vlasveld, E. M. Rankin, A. Hekman, S. Rodenhuis, J. H. Beijnen, A. M. Hilton, A. C. Dubbelman, F. A. Vyth-Dreese, C. J. Melief

**Affiliations:** Department of Medical Oncology, The Netherlands Cancer Institute, Amsterdam.

## Abstract

The optimal schedule for recombinant interleukin-2 (rIL-2) administration is unclear. Because the clinical and immunological effects of prolonged continuous exposure to rIL-2 are unknown, we have conducted a phase I study to assess the toxicity and feasibility of continuous low dose infusion of rIL-2 (EuroCetus) using central venous access with a portable infusion device on an out-patient basis. Twenty-two patients entered the study, 13 with melanoma and nine with renal cell cancer, age range 26-66 years (median 51), performance status less than or equal to 1. They were treated with one of the following doses per m2 per 24 h: 0.18 x 10(6) IU, 0.6 x 10(6) IU, 1.8 x 10(6) IU, 3 x 10(6) IU, 6 x 10(6) IU and 9 x 10(6) IU. Toxicity was evaluable in 20 patients receiving greater than or equal to 3 weeks treatment duration or in whom treatment was discontinued prematurely because of toxicity. Constitutional symptoms consisting of fatigue, malaise and fever up to 40 degrees C without significant organ dysfunction occurred with doses greater than or equal to 1.8 x 10(6) IU m-2. The maximum tolerated dose was 6 x 10(6) IU m-2 24 h-1. In all patients toxicity reached a peak at 3 weeks and resolved thereafter despite continued rIL-2 treatment. Peripheral blood eosinophilia (up to 66% of white blood cell count) followed the same pattern. An infection of the central venous access occurred in 55% of the patients but this was mostly asymptomatic. Thirteen patients were treated greater than or equal to 6 weeks and were evaluable for tumour response. A partial remission occurred in a patient with melanoma with a dose of 1.8 x 10(6) IU rIL-2 m-2 24 h-1.


Br. J. Cancer (1992), 65, 744–750 © Macmillan Press Ltd., 1992

### A phase I study of prolonged continuous infusion of low dose recombinant interleukin-2 in melanoma and renal cell cancer. Part I: clinical aspects

L.T. Vlasveld[1,2], E.M. Rankin[1,2], A. Hekman[2], S. Rodenhuis[1], J.H. Beijnen[3], A.M. Hilton[1], A.C. Dubbelman[1], F.A. Vyth-Dreese[2] & C.J.M. Melief[2]

[1]*Department of Medical Oncology,* [2]*Division of Immunology,* [3]*Pharmacy, The Netherlands Cancer Institute, Plesmanlaan 121 1066 CX Amsterdam, The Netherlands.*

**Summary** The optimal schedule for recombinant interleukin-2 (rIL-2) administration is unclear. Because the clinical and immunological effects of prolonged continuous exposure to rIL-2 are unknown, we have conducted a phase I study to assess the toxicity and feasibility of continous low dose infusion of rIL-2 (EuroCetus) using central venous access with a portable infusion device on an out-patient basis. Twenty-two patients entered the study, 13 with melanoma and nine with renal cell cancer, age range 26–66 years (median 51), performance status $\leqslant 1$. They were treated with one of the following doses per $m^2$ per 24 h: $0.18 \times 10^6$ IU, $0.6 \times 10^6$ IU, $1.8 \times 10^6$ IU, $3 \times 10^6$ IU, $6 \times 10^6$ IU and $9 \times 10^6$ IU. Toxicity was evaluable in 20 patients receiving $\geqslant 3$ weeks treatment duration or in whom treatment was discontinued prematurely because of toxicity. Constitutional symptoms consisting of fatigue, malaise and fever up to 40°C without significant organ dysfunction occurred with doses $\geqslant 1.8 \times 10^6\,IU\,m^{-2}$. The maximum tolerated dose was $6 \times 10^6\,IU\,m^{-2}$ $24\,h^{-1}$. In all patients toxicity reached a peak at 3 weeks and resolved thereafter despite continued rIL-2 treatment. Peripheral blood eosinophilia (up to 66% of white blood cell count) followed the same pattern. An infection of the central venous access occurred in 55% of the patients but this was mostly asymptomatic. Thirteen patients were treated $\geqslant 6$ weeks and were evaluable for tumour response. A partial remission occurred in a patient with melanoma with a dose of $1.8 \times 10^6$ IU rIL-2 $m^{-2}\,24\,h^{-1}$.

The lymphokine interleukin-2 displays a wide range of effects on the immune system. Administration of high-dose *E. coli* -derived recombinant interleukin-2 (rIL-2) as a bolus injection or by continuous infusion may yield remissions in up to 25% of patients with advanced malignant disease, especially malignant melanoma and renal cell cancer (Rosenberg *et al.*, 1987; Negrier *et al.*, 1989). This relatively modest response rate is achieved at the cost of considerable toxicity (Siegel *et al.*, 1991). In an attempt to increase the therapeutic index of rIL-2, a large number of schedules with a variety of dose levels and routes of administration have been explored, and rIL-2 has been combined with *in vitro* expanded lymphokine activated killer (LAK) cells or tumour infitrating lymphocytes, other cytokines, monoclonal antibodies, and various cytostatic and immunomodulating agents (Rosenberg *et al.*, 1989; Truitt *et al.*, 1989; West *et al.*, 1987; Winkelhake *et al.*, 1990). All these manipulations may influence the biological effects of rIL-2, but have not signifcantly improved the therapeutic index.

Treatment with rIL-2 is associated with dose-dependent changes in the immune system. Administration of high-dose rIL-2 may result in significant shifts in lymphocyte (sub)populations, the *in vivo* generation of cells with LAK activity, and enhancement of natural killer (NK) cell activity (Winkelhake *et al.*, 1990). Clinical and experimental data show that antibody-dependent cellular cytotoxicity (ADCC) and the number of activated cytotoxic ($CD8^+$) T lymphocytes (CTL) may be increased after administration of relatively low and less toxic doses of rIL-2 (Hank *et al.*, 1990; Munn *et al.*, 1987; Naito *et al.*, 1988; Talmadge *et al.*, 1987). These HLA class I restricted tumour-specific CTL's are capable of completely eradicating tumours in a variety of experimental murine models and are considered to play an important role in the cellular immunity in human metastatic melanoma (Melief, 1991).

The mechanism(s) of the anti-tumour effect of rIL-2 and the optimal means of administration are unknown. No consistent correlation has been found between the biological effects of rIL-2 and its therapeutic efficacy (Ghosh *et al.*, 1989; Kohler *et al.*, 1989*b*). Although most tumour remissions have been reported after treatment with high-dose rIL-2, objective anti-tumour activity has also been observed with low-dose schedules (Marumo *et al.*, 1989; Stein *et al.*, 1991). These observations and extrapolation from experience with other cytokines such as interferon-γ (Kirkwood *et al.*, 1990) would suggest that the best immuno-modulating dose of rIL-2 is not necessarily equivalent to the maximal tolerated dose. The maximal tolerated dose of rIL-2 has been established in phase I studies exploring a wide range (four logs) of dose levels in various schedules (Creekmore *et al.*, 1989; Kohler *et al.*, 1989*a*). These studies and preclinical models have stressed the role of prolonged elevated serum levels of IL-2 in the stimulation of the immune system (Cheever *et al.*, 1985; Thompson *et al.*, 1988). The serum half-life of rIL-2 is short and continuous infusion or multiple subcutaneous injections are needed to obtain stable serum levels (Thompson *et al.*, 1987). In most reported studies, the period of continuous infusion is 7 days or less, so it is unknown whether prolonged rIL-2 exposure leads to persistent immune stimulation with subsequent improved anti-tumour effect. Between May 1989 and December 1990, we conducted a phase I study to define the feasibility, toxicity and immunological effects of prolonged continuous infusion of rIL-2 at various doses given on an out-patient basis. In this paper we describe the clinical results of this study. The immunological aspects will be discussed in detail elsewhere.

### Materials and methods

### *Patient selection*

Eligible patients ($\geqslant 18$ years old) had progressive, measurable or evaluable, histologically-proven metastatic renal cell cancer or malignant melanoma, good clinical condition (performance status $\leqslant 1$) with a life expectancy of $\geqslant 3$ months, normal bone marrow (leucocytes $\geqslant 4{,}000\,\mu l^{-1}$, platelets $\geqslant 100{,}000\,\mu l^{-1}$), renal (creatinine $\leqslant 1.5\,mg\,dl^{-1}$ or calculated creatinine clearance $\geqslant 60\,ml\,min^{-1}$) and hepatic (bilirubin and alkaline phosphatase $\leqslant 1.5 \times$ upper unit of normal) function, and normal electrocardiogram without clinical signs of cardiac or pulmonary dysfunction. Patients with brain metastases on

Correspondence: L.T. Vlasveld.
Received 19 August 1991; and in revised form 20 December 1991.

CT scan were excluded from the study. Prior radiotherapy (except radiation to a limited area e.g. a painful bone metastasis or localised lymphnode) or chemotherapy within 4 weeks (6 weeks for nitrosoureas or mitomycin C) of study were not allowed and patients were required to have fully recovered from previous therapies. Written informed consent was obtained in accordance with the Netherlands Cancer Institute guidelines. The protocol was approved by the Institute's Ethical Committee.

*Interleukin 2*

The *E. coli*-expressed recombinant rIL-2 was purchased from EuroCetus (Amsterdam, The Netherlands). Unlike human IL-2, EuroCetus rIL-2 is non-glycosylated. The N-terminal alanine is deleted and at position 125 cysteine has been replaced by serine, rIL-2 is provided as lyophilised powder containing 0.2 mg sodium dodecyl sulphate and 50 mg mannitol per $3 \times 10^6$ Units (= $18 \times 10^6$ International Units (IU)). [See Note at end of paper].

The rIL-2 was reconstituted as previously described with 1.2 ml sterile water and diluted in sterile water containing 2% human serum albumin until the required rIL-2 concentration had been achieved (Vlasveld *et al.*, 1990). The solution was aseptically prepared by the Pharmacy department and delivered in 12 ml plastic syringes (Monoject, Sherwood, St Louis, USA) which were stored at $-20°C$ for a maximum of 7 days until use.

*Infusion device*

Central venous access was obtained via a Vascuport (Viggo, Leusden, The Netherlands) consisting of an implantable titanium injection portal with an 11.5 mm thick silicone membrane and a polyurethane catheter of variable length (inner diameter 1.20 mm). The Perfusor M (Braun, Uden, The Netherlands) was used as a portable pump; this device empties a 10 ml syringe in 24 h. The pump and the Vascuport were connected by a 200–300 cm long polyethylene catheter with an inner diameter of 1.5 mm (Lectro-cath) (Vygon, Veenedaal, the Netherlands).

*Study design*

The study was designed to define the feasibility, toxicity, and immunomodulatory effects of continuous infusion of rIL-2 given on an out-patient basis. Within a fortnight before entry to the study a history and full physical examination and the following investigations were performed, complete blood count with differential, an extensive chemistry profile, urinalysis, electrocardiogram, chest radiograph, and appropriate radiologic studies to evaluate the presence of measurable disease. The central venous catheter was inserted under general anaesthesia. Treatment with rIL-2 was started within 1 week of this operation. After discharge from the hospital the patient was seen weekly at the out-patient clinic. During treatment, clinically accessible lesions were measured and chest X ray repeated at 3 weekly intervals. Computed tomography or ultrasound scans were repeated every 6 weeks. Before, every 3 weeks during treatment, and 2–4 weeks after treatment, 30 ml peripheral blood was taken, the lymphocytes were isolated by centrifugation over Ficoll, and then stored in liquid nitrogen. When possible, weekly blood cultures were taken from the central venous catheter.

Recombinant IL-2 was infused continuously for 24 h a day, 7 days a week. The syringe containing the daily-dosage of rIL-2 was thawed at room temperature and then placed in the perfusor pump by the patient using aseptic technique. The six dose levels $0.18 \times 10^6$, $0.6 \times 10^6$, $1.8 \times 10^6$, $3 \times 10^6$, $6 \times 10^6$ and $9 \times 10^6$ IU $m^{-2}$ 24 $h^{-1}$ were tested to define toxicity. Patients were considered evaluable for toxicity, if rIL-2 had been continuously administered for 3 weeks or if the treatment was prematurely discontinued because of toxicity. At least three evaluable patients were entered at each dose level. Because the three highest dose levels were mainly explored to determine the maximal tolerated dose, intrapatient dose escalation was permitted in those patients. In addition, the four dose levels $0.18 \times 10^6$, $0.6 \times 10^6$, $1.8 \times 10^6$ and $6 \times 10^6$ IU $m^{-2}$ 24 $h^{-1}$ were explored for immunological effects. Patients were considered evaluable for immunomodulatory effects providing they had received the rIL-2 continuously for a period of 6 weeks without any interruption. In this group of patients intrapatient dose escalation, previous rIL-2 treatment or concomitant administration of nonsteroidal anti-inflammatory drugs were not allowed.

*Response criteria*

A complete response was defined as the complete disappearance of all objective evidence of disease for a minimum of 4 weeks. A partial response was defined as a decrease of $\geqslant 50\%$ in the sum of the product of the longest perpendicular diameter of all measured lesions, no progression of evaluable disease, and no new lesions for at least 4 weeks. Progressive disease was defined as an increase of 25% of measured lesions, or the development of new lesions. Lesions, that had been irradiated within 4 weeks before entry of the study were not evaluable for response. Patients were evaluated for response at 3–6 week intervals and response duration was calculated from the date of the initial response.

*Toxicity*

Toxicity was scored weekly according to the World Health Organization (WHO) criteria. For the side-effects for which no standard WHO toxicity criteria are available the following scoring system was used: for malaise and fatigue: grade 1 = mild, no impairment of daily activity; grade 2 = moderate impairment of daily activity; grade 3 = severe, $<50\%$ bedridden during waking hours; grade 4 = intolerable, $>50\%$ bedridden during waking hours: For myalgia/arthalgia: grade 1 = mild, no use of analgesics; grade 2 = moderate, occasional use of analgesics; grade 3 = severe, constant use of anglesics; grade 4 = intolerable, bedridden and constant use of analgesics. Anaemia was defined as the decrease of the haemoglobin level (mmol $l^{-1}$) in comparison with the pretreatment level: grade 1 = decrease of 1–2 mmol $l^{-1}$; grade 2 = decrease 2–3 mmol $l^{-1}$; grade 3 = decrease of 3–4 mmol $l^{-1}$; grade 4 = decrease of $>4$ mmol $l^{-1}$.

The maximum tolerable dose was defined as the dose below that dose at which more than one third of the patients treated experienced dose limiting toxicity. Because the treatment was designed as an out-patient therapy, any toxicity $\geqslant$grade 3 was considered dose limiting.

*Supportive care*

Paracetamol was given at a maximum dose of $6 \times 500$ mg to relieve constitutional symptoms. Nausea and vomiting were treated with metoclopramide 10–20 mg. The use of prostaglandin-inhibitors was avoided in the patients who were evaluated for immunomodulatory effects.

*Statistical analysis*

The results were compared using the Student *t*-test of paired samples. A $P$ value of $\leqslant 0.05$ was considered statistically significant.

### Results

*Patients characteristics*

Twenty-two patients entered the study. The patient characteristics are summarised in Table I. A total of 30 cycles (i.e. period of continuous rIL-2 administration) were administered in these 22 patients as indicated in Table II. Fifteen patients received only one cycle. Treatment was stopped in these patients because of tumour progression (12), toxicity (one), CVA infection (one) and patient refusal (one). Seven patients received a total of 15 separate cycles. Treatment was stopped prematurely (<3 weeks) in ten of these cycles because toxicity (four), CVA infection (four) and early tumour progression (two). In four patients the dose was escalated, in three cases from $3 \times 10^6$ to $6 \times 10^6$ IU m$^{-2}$ (in two patients after 3 weeks and in one patient after 7 weeks) and in one patient after 10 weeks at $6 \times 10^6$ IU m$^{-2}$ the dose was escalated to $9 \times 10^6$ IU m$^{-2}$ 24 h$^{-1}$.

**Table I** Patients characteristics

| | |
|---|---|
| *Sex* | |
| Male | 12 |
| Female | 10 |
| *Age* | |
| median | 51 |
| range | 26–66 |
| *Performance* | |
| 0 | 19 |
| 1 | 3 |
| *Diagnosis* | |
| melanoma | 13 |
| renal cell cancer | 9 |
| *Pretreatment*[a] | |
| surgery | 20 (nephrectomy 8) |
| radiotherapy | 6 |
| chemotherapy | 3 |
| melphalan limb perfusion | 3 |
| none | 2 |
| *Interval since initial diagnosis (months)* | |
| median | 34 |
| range | 2–168 |
| *Interval since last treatment (weeks)*[a] | |
| median | 10.5 |
| range | 1–156 |
| *Tumour sites* | |
| cutaneous | 3 |
| pulmonary | 9 |
| lymphnode | 6 |
| bone | 4 |
| hepatic | 3 |
| miscellaneous | 5 |

[a]In total three patients were treated <4 weeks prior to entry of the study. Nephrectomy was performed in one patient and in two patients a (sub)cutaneous metastasis was removed.

**Table II** Characteristics of 30 administered cycles of rIL-2 in 22 patients

| | | |
|---|---|---|
| Duration <3 weeks | 10 | |
| Dose levels (IU m$^{-2}$) | | |
| $3 \times 10^6$ | | 3 |
| $6 \times 10^6$ | | 5 |
| $9 \times 10^6$ | | 2 |
| Reason for discontinuation | | |
| CVA infection | | 4 |
| Toxicity | | 4 |
| Early progression | | 2 |
| Duration 3–6 weeks | 3 | |
| Dose levels (IU m$^{-2}$) | | |
| $0.6 \times 10^6$ | | 1 |
| $6 \times 10^6$ | | 2 |
| Reason for discontinuation | | |
| Early progression | | 3 |
| Duration >6 weeks | 17 | |
| Dose levels (IU m$^{-2}$) | | |
| $0.18 \times 10^6$ | | 3 |
| $0.6 \times 10^6$ | | 2 |
| $1.8 \times 10^6$ | | 3 |
| $3 \times 10^6$ | | 4 |
| $6 \times 10^6$ | | 4 |
| $9 \times 10^6$ | | 1 |
| Reason for discontinuation | | |
| CVA infection | | 2 |
| Toxicity | | 2 |
| Progression | | 11 |
| No change (patient refusal) | | 2 |

CVA = central venous access.

### *Feasibility*

The patients quickly learned to manage the pump and to replace the syringes and were discharged from the hospital after 3–4 days of training. Slow rate continuous infusion for a prolonged period of time via central venous access (CVA) was frequently complicated by infusion interruption and infection of the CVA. These complications mainly occurred during the first period of the study, but decreased both in number and severity as the study continued with better attention to technique and rigorous hygiene. A mechanical defect of the portable pumps occurred only once during the total of 1,336 days of use. Especially during the first weeks of treatment, interruption of the infusion occurred regularly and was mainly due to mismanagement of the pump or to dislocation of the insertion needle in the port of the CVA. Catheter obstruction occurred in three cases and was associated with thrombus formation at the tip of the CVA catheter in only one case. In one case the infusion line was accidently cut in half by the patient while trimming the garden hedge and in another case the line was bitten through by the family pet. For the patient the main discomfort consisted of the constant confrontation with the disease, impaired activities of daily living e.g. inability to take a shower, and impeded sex-life. In no patient did this inconvenience prompt discontinuation of the treatment.

### *Toxicity*

Twenty patients who had continuous treatment with rIL-2 for ≥3 weeks were evaluable for toxicity. The duration of treatment ranged from 21 to 126 days (median 50). In one of the 22 entered patients the administration of rIL-2 was discontinued at <3 weeks because of toxicity, and in another patient treatment was stopped prematurely because of the occurrence of epidural metastasis.

The toxicity is shown in Tables IIIa and b. In the four cases of intrapatient dose escalation, the toxicity of the lower dose level is scored in these tables. Toxicity was dose dependent. In doses up to $3 \times 10^6$ IU m$^{-2}$ treatment was well tolerated with mild side effects consisting of a mild flu-like syndrome with malaise, fatigue, myalgia and low grade fever, runny nose and erythema.

**Table IIIa** Clinical toxicity of continuous low dose rIL-2 administered for ≥3 weeks

| Symptom | *0.18* *n = 3* | *0.6* *n = 3* | *1.8* *n = 3* | *3* *n = 4* | *6* *n = 6* | *9* *n = 1* |
|---|---|---|---|---|---|---|
| Fever | | | | | | |
| gr 1 | – | – | 1 | – | 1 | – |
| gr 2 | – | 1[b] | – | 1 | 3 | 1 |
| Chills | | | | | | |
| gr 1 | – | – | – | – | 2 | – |
| gr 2 | – | – | – | – | – | 1 |
| Malaise/fatigue | | | | | | |
| gr 1 | – | 3 | – | 1 | – | – |
| gr 2 | – | – | 1[b] | 1 | 6 | – |
| gr 4 | – | – | – | – | – | 1 |
| Myalgia/arthralgia | | | | | | |
| gr 1 | – | – | 1 | 2 | 3 | 1 |
| gr 2 | – | – | – | – | 3 | – |
| Nausea/vomiting | | | | | | |
| gr 1 | – | – | 1 | – | 2 | – |
| gr 2 | – | – | – | – | 2 | 1 |
| gr 3 | – | – | – | – | 1 | – |
| Runny nose | – | – | – | 2 | 3 | 1 |
| Erythema | | | | | | |
| gr 1 | – | 1 | – | 2 | 1 | – |
| gr 2 | – | – | – | – | 1 | 1 |
| Cheilitis | | | | | | |
| gr 1 | – | 2 | – | – | 2 | 1 |
| Hypotension | | | | | | |
| gr 2 | – | – | – | 1[d] | – | – |

Dose rIL-2 × $10^6$ m$^{-2}$ 24 h$^{-1}$. See also footnote at the end of Table IIIb.

**Table IIIb** Laboratory toxicity of continuous low dose rIL-2 administered for ≥3 weeks

| *Symptom* | *0.18* *n = 3* | *0.6* *n = 3* | *1.8* *n = 3* | *3* *n = 4* | *6* *n = 6* | *9* *n = 1* |
|---|---|---|---|---|---|---|
| Haemoglobin (mmol $l^{-1}$) | | | | | | |
| gr 1 | – | – | – | – | 1 | – |
| gr 2 | – | – | – | – | 2 | 1 |
| gr 3 | – | 1[c] | 1[c] | – | – | – |
| Serum creatinine | | | | | | |
| gr 1 | – | – | – | 1[d] | – | – |
| gr 2 | – | – | 1[c] | – | 1[e] | – |
| gr 4 | – | – | – | – | – | 1 |
| Hepatic dysfunction | | | | | | |
| gr 1 | – | 1 | – | – | 1 | – |
| gr 2 | – | – | – | 1 | – | 1 |
| gr 3 | – | – | – | – | 2 | – |
| CVA infection[a] | 2 | 1 | 2 | 4 | 7 | 1 |

Dose rIL-2 × $10^6$ $m^{-2}$ 24 $h^{-1}$. Table III shows the clinical (Table IIIa) and laboratory (Table IIIb) toxicity of continuous infusion of various doses of rIL-2 according to the standard WHO criteria. For side effects for which no standard WHO criteria are available, the toxicity score is defined in the text. The haemoglobin level (mmol $l^{-1}$) was compared with the pretreatment level. [a]CVA infection = separate episodes of positive blood cultures drawn from the central venous access. [b]Tumour related, [c]related to tumour progression, [d]related to concomitant use of antihypertensive drugs, [e]related to concomitant use of NSAID.

All patients with 6 × $10^6$ IU $m^{-2}$ 24 $h^{-1}$ rIL-2 experienced a pronounced flu-like syndrome with fatigue, myalgia, fever up to 40°C and chills, nausea, vomiting and erythema. These symptoms could be easily managed with simple supportive measures. As indicated in Table II a total number of 11 cycles of 6 × $10^6$ IU $m^{-2}$ has been given. In three patients the tretament was disrupted because of subjective toxicity consisting of grade 2 malaise/fatigue and grade 2 fever. In two of these patients who had received treatment for 5 and 27 days, rIL-2 treatment was reinstituted at the same dose level. In one patient the administration of rIL-2 was discontinued after a further 10 days because of persistent fever with chills and tumour progression while the other patient completed a total 6 weeks course after restarting with only grade 1 toxicity. In the third patient treatment was stopped after 19 days and not reinstituted because of possible rIL-2-related mental exhaustion and depression.

Three patients were treated with 9 × $10^6$ IU $m^{-2}$ 24 $h^{-1}$ rIL-2. Two patients experienced high grade fever up to 41°C with chills, diffuse erythema and severe fatigue so that the patient was bedridden. Treatment was discontinued after 3 and 4 days respectively. The third patient was treated for a period of 6 weeks tolerating severe constitutional symptoms. During the 6th week treatment was stopped because of acute renal failure due to an acute interstitial nephritis which resolved after haemodialysis and prednisone pulse therapy (Diekman *et al.*, 1992).

Interestingly, toxicity first because manifest within 3–5 days of treatment reached a peak after 2–3 weeks of treatment and subsequently decreased despite continuation of the rIL-2 treatment. In the three patients in whom the dose was escalated from 3 × $10^6$ to 6 × $10^6$ IU $m^{-2}$ 24 $h^{-1}$ and in the patient in whom the dose was escalated from 6 × $10^6$ to 9 × $10^6$ IU $m^{-2}$ 24 $h^{-1}$ only mild and transient increase in toxicity was observed following the dose escalation. Apart from the case of acute interstitial nephritis, organ dysfunction was clinically insignificant. Hepatic toxicity consisted of elevation of the transaminases and alkaline phosphatase without hyperbilirubinemia and occurred mostly during the first 3 weeks of treatment and resolved despite continuation of the rIL-2 treatment. Deterioration in renal function occurred in four patients and was related in individual patients to the concomitant use of antihypertensive drugs (leading to hypotension grade 2), non-steroidal anti-inflammatory drug (NSAID), tumour progression or possible rIL-2 related-interstitial nephritis. Cardiac and pulmonary dysfunction or signs of capillary leakage were not observed. No changes in blood pressure were observed, except in one patient who was concomitantly treated with antihypertensive drugs.

Skin toxicity consisted of diffuse or local erythema especially on palms or soles with subsequent desquamation, folliculitis on the trunk, occasionally subtle nail changes and once localised bulla at the site of a peripheral vein access. Oral and mucosal toxicity included cheilitis, change in taste and dryness of oral and vaginal mucosa. Some patients complained of emotional lability, impotence or decreased libido. All these symptoms occurred at the higher dose levels, were transient and of minor clinical importance.

The maximum tolerated dose for this type of treatment was 6 × $10^6$ IU $m^{-2}$ 24 $h^{-1}$ and consisted of moderate malaise, fatigue and fever up to 40°C.

### *Haematological effects*

Except for the occurrence of anaemia in patients treated at the highest dose, no haematological toxicity was encountered. There were no significant changes in the lymphocyte and granulocyte counts. However, there was a dose dependent increase in the eosinophil counts as indicated in Figure 1a–f and Table IV. At the higher dose levels the eosinophils increased to approximately 20,000 $\mu l^{-1}$ making up two thirds of the total white blood count. The eosinophil count reached a peak around the second and third week of treatment and decreased thereafter despite continuation of the rIL-2. Intrapatient dose escalation, performed at the highest dose levels, did not result in an additional increase or second peak in the eosinophil count. No changes in the prothrombin time and partial thromboplastin time were observed.

### *Immunological effects*

The effects on the peripheral blood lymphocytes will be discussed in detail in a separate paper. In summary, no effects were seen in patients treated with 0.18 and 0.6 × $10^6$ IU $m^{-2}$ 24 $h^{-1}$. At the doses of 1.8 and 6 × $10^6$ IU $m^{-2}$ 24 $h^{-1}$ the percentage of natural killer cells (NK) increased to up to 75% of the peripheral blood lymphocytes. In addition there was an increased number of cells expressing CD25 (α chain IL-2 receptor), p75 (β chain IL-2 receptor), and CD38. An increase in activated NK activity and in the number of LAK precursor cells were observed. Furthermore, antibody dependent cellular cytotoxicity (ADCC) was enhanced. In contrast to the toxic and haematological effects these immunological effects persisted throughout the entire treatment period.

### *Infections*

An infection of the CVA was defined as the presence of a positive culture in blood drawn from the CVA in symptomatic as well as asymptomatic patients. In symptomatic patients (fever >38°C and/or chills) and in some of the asymptomatic patients peripheral blood cultures were obtained after a CVA infection had been documented. Sixteen episodes of CVA infection were noted in 12 of 22 patients (55%) (11 × *Staphylococcus epidermidis*, 1 × *Pseudomonas maltophilia*, 1 × *Bacillus*, 1 × *Klebsiella pneumoniae*, 1 × *Corynebacterium*, 1 × *Pseudomonas species*). Nine of these CVA infections occurred in the first eight patients treated. Infection was documented on average 25 days after the start of the treatment (range 5–79). The presenting symptoms were fever and chills (six) or local infection at the entry site (one), nine infections were asymptomatic. The infection prompted interruption of the rIL-2 treatment in six cases and in two cases the CVA was removed. The infections were treated with systemic antibiotic treatment through the CVA in ten cases. As the study progressed the asymptomatic CVA infections were considered as bacterial colonisation of the catheter and were not treated in six cases.

In two of the total of six symptomatic episodes of CVA infections peripheral blood cultures were positive (*Staphylococcus epidermidis, Bacillus*). Two additional cases of CVA infections occurred before the start of rIL-2 treatment.

**Figure 1(a–f)** Changes in the mean leukocyte, lymphocyte and eosinophil counts during treatment with rIL-2. At doses $\geqslant 1.8 \times 10^6$ IU m$^{-2}$ 24 h$^{-1}$ a transient and significant rise in the eosinophil count occurred. -■- leucocytes, -●- eosinophils, -O- lymphocyte.

**Table IV** Effects of continuous infusion of rIL-2 on eosinophil count

| *Weeks* | *Mean (cells µl$^{-1}$)* | *s.d.* | *P value*[a] |
|---|---|---|---|
| pre | 210 | 138 | |
| 1 | 290 | 117 | n.s. |
| 2 | 2980 | 3254 | n.s. |
| 3 | 4770 | 3885 | n.s. |
| 4 | 3270 | 2060 | n.s. |
| 5 | 3620 | 1729 | n.s. |
| 6 | 1530 | 1225 | n.s. |

Dose rIL-2: $1.8 \times 10^6$ IU m$^{-2}$ 24 h$^{-1}$. ($n = 3$)

| *Weeks* | *Mean (cells µl$^{-1}$)* | *s.d.* | *P value*[a] |
|---|---|---|---|
| pre | 230 | 236 | |
| 1 | 340 | 466 | n.s. |
| 2 | 2680 | 1766 | n.s. |
| 3 | 5450 | 4767 | n.s. |
| 4 | 3230 | 1997 | n.s. |
| 5 | 3000 | 1901 | n.s. |
| 6 | 2050 | 616 | <0.05 |

Dose rIL-2: $6 \times 10^6$ IU m$^{-2}$ 24 h$^{-1}$. ($n = 3$).

The total eosinophil count was determined before and at weekly intervals during treatment. [a]Student (paired) $t$ test, comparing the pre-treatment with levels during treatment. s.d. = standard deviation.

### *Thrombotic complications*

In 15 of the total of 18 episodes of CVA infection, and in three cases of catheter obstruction without infection the patency of the CVA was examined radiologically following injection of contrast into the CVA. Evidence of thrombus formation near the tip of the catheter was present in six cases of CVA infection (5 × culture proven, 1 × strong clinical suspicion) and in one case of flow obstruction. In one of these seven thrombotic events occlusion of the right brachiocephalic vein was present.

In six cases the thrombus resolved within 24–48 h following low dose streptokinase infusion via the CVA. The venous occlusion was successfully treated with systemic recombinant tissue plasminogen activator (rTPA, Boehringer-Ingelheim, Alkmaar, The Netherlands).

### *Responses*

Thirteen patients could be evaluated for anti-tumour response after at least 6 weeks of uninterrupted rIL-2 treatment. Of the patients with malignant melanoma four had stable disease, three had progression and one achieved partial remission in a lymphnode metastasis. The partial remission occurred with a dose at $1.8 \times 10^6$ IU m$^{-2}$ and was noted within the first 3 weeks of treatment and lasted for 5 weeks. In patients with renal cell cancer four patients had stable disease and one had progression.

### Discussion

This phase I study demonstrates that continuous self-administration of rIL-2 by a central venous access (CVA) for a prolonged period of time on an out-patient basis is feasible. The CVA-related complications such as infusion interruption and CVA infection occurred especially during the first part of the study and decreased both in number and severity as the experience with this mode of treatment increased. In total 16, mostly asymptomatic, infections of the central venous access were noted in 12 of the 22 patients treated with rIL-2 (55%) and these data are in accordance with the observed high incidence of line and systemic infections with predominantly gram-positive cocci in patients treated with high-dose rIL-2 (Clark *et al.*, 1990; Snydman *et al.*, 1990). Although we did not find a correlation between the incidence of infection and the dose of rIL-2, the high infection rate may be related to the known reversible effect of rIL-2 on the chemotaxis of the neutrophils (Klempner *et al.*, 1990) in combination with the very slow infusion of a protein-rich solution for a long period of time. In approximately one third of the infections of the central venous access, a thrombus at the tip of the catheter, without clinical signs of flow obstruction, could be demonstrated. The clinical significance of the thrombus formation is at present uncertain. We decided to resolve the clots to avoid occlusion of major veins. Local infusion of low-dose streptokinase was successful in 100% of cases and was without untoward effects.

The systemic toxicity in this phase I study was dose dependent. No toxicity was observed up to a dose of $0.6 \times 10^6$ IU $m^{-2}$ 24 $h^{-1}$. At doses of $1.8 \times 10^6$, $3 \times 10^6$, and $6 \times 10^6$ IU $m^{-2}$ 24 $h^{-1}$, toxicity mainly consisted of easily manageable constitutional symptoms without significant organ dysfunction or vascular leakage. At a dose of $9 \times 10^6$ IU $m^{-2}$ 24 $h^{-1}$, grade 4 toxicity occurred and prompted discontinuation of treatment. Based on the presence of moderate fatigue/malaise and fever $\leqslant 40°C$ the maximal tolerated dose was defined as $6 \times 10^6$ IU $m^{-2}$ 24 $h^{-1}$ for this mode of treatment on an outpatient basis.

In most reported studies of continuous infusion of rIL-2, the duration of administration varies from 1 to 5 days per week. Depending on the treatment schedule the reported maximal tolerated dose varies from $12 \times 10^6$ U $m^{-2}$ $day^{-1}$ (Cetus) or $30 \times 10^6$ U $m^{-2}$ $day^{-1}$ (Hoffmann-LaRoche) for 24 h infusion to $1 \times 10^6$ U $m^{-2}$ $day^{-1}$ (Hoffmann-LaRoche) or 1,000 U $kg^{-1}$ $h^{-1}$ ($\approx 1 \times 10^6$ U $m^{-2}$ $day^{-1}$) (Cetus) for $\geqslant 1$ week treatment (Creekmore *et al.*, 1989; Kohler *et al.*, 1989*a*; Lotze *et al.*, 1985; Perez *et al.*, 1991) [see Note].

Rest days to recover from the toxicity are often needed. In one of the earliest phase I studies, Lotze *et al.* gave rIL-2 by continuous infusion for a period of 2–3 weeks at a maximal tolerated dose of $1 \times 10^6$ U $m^{-2}$ 24 $h^{-1}$ (Cetus) in two patients (Lotze *et al.*, 1985; 1987). In contrast to the findings in the present study, the dose limiting toxicity consisted of vascular leakage syndrome. Recently, two studies with prolonged daily administrations of low dose rIL-2 (100 $\mu g$ $day^{-1}$, Glaxo/Biogen) as short term twice-daily intravenous infusion or by subcutaneous injection for up to 80 days have been reported with toxicity and immunological effects which are comparable with those found by us (Marumo *et al.*, 1989; Stein *et al.*, 1991). Interestingly, in the present study, signs of toxicity appeared 3–5 days after initiation of treatment, reached a peak at 2–3 weeks and decreased afterwards despite continuation of the treatment. This phenomenon was also observed by Lotze, but was not reported by Stein and Marumo. This pattern was also noted with regard to the changes in the eosinophil counts. The rIL-2 related toxicity has been ascribed to the release of secondary cytokines such as interferon and tumour necrosis factor (Gemlo *et al.*, 1988). The effect on the eosinophil count is considered to be the result of the release of interleukin 5 (Macdonald *et al.*, 1990). In theory, several mechanisms may account for the transience of the biological effects of rIL-2 such as the occurrence of neutralising antibodies against rIL-2, down-regulation of one or both components of the rIL-2 receptor, the generation or up-regulation of inhibitory or negative feedback mechanisms, or 'exhaustion' of the production or release of the secondary cytokines. In view of the discrepancy between the transient toxic and haematologic effects, and the observed persistent immunological enhancement and upregulation of the IL-2 receptor, failure of the secondary cytokine mechanisms may be the most likely.

The antitumour effect of the regimen used in this phase I study consisted of an objective partial remission in a patient with a lymphnode metastasis from a malignant melanoma. This brief partial remission occurred in one of the three patients who were treated with the well-tolerated dose of $1.8 \times 10^6$ IU $m^{-2}$ 24 $h^{-1}$. Interestingly this tumour regression occurred during the first 3 weeks of treatment which was continued until a new lesion developed 5 weeks later. This lesion was excised and the lymphnode metastasis disappeared in the subsequent months. One year after the end of the rIL-2 treatment the patient developed multiple brain metastases. In the other two patients treated at this dose a minimal tumour regression was seen during the first 3 weeks with subsequent tumour progression despite further treatment.

Based on these observations phase II studies have been initiated of intermittent continuous infusion for a period of 3 weeks at a dose of $1.8 \times 10^6$ IU $m^{-2}$ 24 $h^{-1}$ in patients with malignant melanoma or renal cell cancer.

**Note:**

The specific activity and the unitage of the various rIL-2 compounds differ considerably: (Euro)Cetus (Proleukin®): $3 \times 10^6$ U $mg^{-1}$ protein, Hoffmann-LaRoche (Teceleukin®): $1.5 \times 10^7$ U $mg^{-1}$ protein, Glaxo/Biogen (Bioleukin®): $1.7 \times 10^6$ U $mg^{-1}$ protein (Stein *et al.*, 1991) – $1 \times 10^7$ U $mg^{-1}$ protein (Marumo *et al.*, 1989). The interim reference reagent (established in 1984) prepared from a human T cell line and containing a nominal 500 Biological Response Modifiers Program Units has been standardised in 1987 by the World Health Organization with a defined potency of 100 International Units (IU = Biological Response Modification Project Interim International Reference Standard Unit) (Gearing *et al.*, 1988). As a consequence one Cetus Unit equals six International Units (Perez *et al.*, 1991) and one Hoffmann-LaRoche Unit equals one International Unit (Creekmore *et al.*, 1989).

### References

CHEEVER, M.A., THOMPSON, J.A., KERN, D.E. & GREENBERG, P.D. (1985). Interleukin 2 (IL 2) administered *in vivo*: influence of Il 2 route and timing on T cell growth. *J. Immunol.*, **134**, 3895.

CLARK, J.W., SMITH II, J.W., STEIS, R.S. & 13 others (1990). Interleukin 2 and lymphokine-activated killer cell therapy: analysis of a bolus interleukin 2 and a continuous infusion interleukin 2 regimen. *Cancer Res.*, **50**, 7343.

CREEKMORE, S.P., HARRIS, J.E., ELLIS, T.M. & 7 others (1989). A phase I clinical trial of recombinant interleukin-2 by periodic 24-hour intravenous infusion. *J. Clin. Oncol.*, **7**, 276.

DIEKMAN, M.J.M., VLASVELD, L.T., KREDIET, R.T., RANKIN, E.M. & ARISZ, L. (1992). Acute interstitial nephritis during continous intravenous administration of low-dose interleukin-2. *Nephron*, (in press).

GEARING, A.J.H. & THORPE, R. (1988). The international standard for human interleukin-2: calibration by international collaborative study. *J. Immunol. Meth.*, **144**, 3.

GEMLO, B.T., PALLADINO, M.A., JAFFE, H.S., ESPEVIK, T.P. & RAYNER, A.A. (1988). Circulating cytokines in patients with metastatic cancer treated with recombinant interleukin 2 and lymphokine-activated killer cells. *Cancer Res.*, **48**, 5864.

GHOSH, A.K., DAZZI, H., THATCHER, N. & MOORE, M. (1989). Lack of correlation between peripheral blood lymphokine-activated killer (LAK) cell function and clinical response in patients with advanced malignant melanoma receiving recombinant interleukin 2. *Int. J. Cancer*, **43**, 410.

HANK, J.A., ROBINSON, R.R., SURFUS, J. & 4 others (1990). Augmentation of antibody dependent cell mediated cytotoxicity following *in vivo* therapy with recombinant interleukin 2. *Cancer Res.*, **50**, 5234.

KIRKWOOD, J.M., LOGAN, T.F., VLOCK, D.R. & ERNSTOFF, M.S. (1990). Biological response modifiers in the therapy of metastatic melanoma. In *Therapy of Advanced Melanoma*, Rümke, P. (ed.). Pigment cell vol. 10, p. 105. Karger: Basel.

KLEMPNER, M.S., NORING, R., MIER, J.W. & ATKINS, M.B. (1990). An acquired chemotactic defect in neutrophils from patients receiving interleukin-2 immunotherapy. *N. Engl. J. Med.*, **322**, 959.

KOHLER, P.C., HANK, J.A., MOORE, K.H. & 4 others (1989*a*). Phase 1 clinical trial of recombinant interleukin-2: a comparison of bolus and continuous intravenous infusion. *Cancer Invest.*, **7**, 213.

KOHLER, P.C. & SONDEL, P.M. (1989*b*). The role of interleukin-2 in cancer therapy. *Cancer Surv.*, **8**, 861.

LOTZE, M.T., MATORY, Y.L., ETTINGHAUSEN, S.E. & 5 others (1985). *In vivo* administration of purified human interleukin 2: II: Half life, immunologic effects, and expansion of peripheral lymphoid cells *in vivo* with recombinant IL 2. *J. Immunol.*, **135**, 2865.

LOTZE, M.T., CUSTER, M.C., SHARROW, S.O., RUBIN, L.A., NELSON, D.L. & ROSENBERG, S.A. (1987). *In vivo* administration of purified human interleukin-2 to patients with cancer: development of interleukin-2 receptor positive cells and circulating soluble interleukin-2 receptors following interleukin-2 administration. *Cancer Res.*, **47**, 2188.

MACDONALD, D., GORDON, A.A., KAJITANI, H., ENOKIHARA, H. & BARRETT, A.J. (1990). Interleukin-2 treatment-associated eosinophillia is mediated by interleukin-5 production. *Br. J. Haematol.*, **76**, 168.

MARUMO, K., MURAKI, J., UENO, M. & 6 others (1989). Immunologic study of human recombinant interleukin-2 (low-dose) in patients with advanced renal cell carcinoma. *Urology*, **33**, 219.

MELIEF, C.J.M. (1991). Tumor eradication by adoptive transfer of cytotoxic T lymphocytes. In *Adoptive Immunotherapy*, Klein, G. & VandeWoude, G. (eds). Advances in Cancer Research. Academic Press; Orlando. (in press).

MUNN, D.H. & CHEUNG, N.-K.V. (1987). Interleukin-2 enhancement of monoclonal antibody-mediated cellular cytotoxicity against human melanoma. *Cancer Res.*, **47**, 6600.

NAITO, K., PELLIS, N.R. & KAHAN, B.D. (1988). Effect of continuous administration of interleukin-2 on active specific chemoimmunotherapy with extracted tumour-specific transplantation antigen and cyclophosphamide. *Cancer Res.*, **48**, 101.

NEGRIER, S., PHILIP, T., STOTER, G. & 16 others (1989). Interleukin-2 with or without LAK cells in metastatic renal cell carcinoma: a report of a European multicentre study. *Eur. J. Cancer Clin. Oncol.*, **25** (Suppl 3), 21.

PEREZ, E.A., SCUDDER, S.A., MEYERS, F.A., TANAKA, M.S., PARADISE, C. & GANDARA, D.R. (1991). Weekly 24-hour continuous infusion interleukin 2 for metastatic melanoma and renal cell carcinoma: a phase I study. *J. Immunother.*, **10**, 57.

ROSENBERG, S.A., LOTZE, M.T., MUUL, L.M. & 10 others (1987). A progress report on the treatment of 157 patients with advanced cancer using lymphokine-activated killer cells and interleukin-2 or high-dose interleukin-2 alone. *N. Engl. J. Med.*, **316**, 889.

ROSENBERG, S.A., LOTZE, M.T., YANG, J.C. & 4 others (1989). Experience with the use of high-dose interleukin-2 in the treatment of 652 cancer patients. *Ann. Surg.*, **210**, 474.

SIEGEL, J.P. & PURI, R.K. (1991). Interleukin-2 toxicity. *J. Clin. Oncol.*, **9**, 694.

SNYDMAN, D.R., SULLIVAN, B., GILL, M., GOULD, J.A., PARKINSON, D.R. & ATKINS, M.B. (1990). Nosocomial sepsis associated with interleukin-2. *Ann. Intern. Med.*, **112**, 102.

STEIN, R.C., MALKOVSKA, V., MORGAN, S. & 8 others (1991). The clinical effects of prolonged treatment of patients with advanced cancer with low-dose subcutaneous interleukin-2. *Br. J. Cancer*, **63**, 275.

TALMADGE, J.E., PHILLIPS, H., SCHINDLER, J., TRIBBLE, H. & PENNINGTON, R. (1987). Systemic preclinical study on the therapeutic properties of recombinant human interleukin 2 for the treatment of metastatic disease. *Cancer Res.*, **47**, 5725.

THOMPSON, J.A., LEE, D.J., COX, W.W. & 5 others (1987). Recombinant interleukin 2 toxicity, pharmacokinetics, and immunomodulatory effects in a phase I trial. *Cancer Res.*, **47**, 4202.

THOMPSON, J.A., LEE, D.J., LINDGREN, C.G. & 4 others (1988). Influence of dose and duration of infusion of interleukin-2 on toxicity and immunomodulation. *J. Clin. Oncol.*, **6**, 669.

TRUITT, G.A., BRUNDA, M.J., LEVITT, D., ANDERSON, T.D. & SHERMAN, M.I. (1989). The therapeutic activity in cancer of IL-2 in combination with other cytokines. *Cancer Surv.*, **8**, 875.

VLASVELD, L.T., RANKIN, E.M., RODENHUIS, S. & 5 others (1990). Reconstitution of interleukin-2. *Lancet*, **336**, 446.

WEST, W.H., TAUER, K.W., YANELLI, J.R. & 4 others (1987). Constant-infusion recombinant interleukin-2 in adoptive immunotherapy of advanced cancer. *N. Engl. J. Med.*, **316**, 898.

WINKELHAKE, J.L. & GAUNY, S.S. (1990). Human recombinant interleukin-2 as an experimental therapeutic. *Pharmacol. Rev.*, **42**, 1.